# How Brain Infarction Links With the Microbiota–Gut–Brain Axis: Hints From Studies Focusing on the Risk Factors for Ischemic Stroke

**DOI:** 10.3389/fnins.2022.877937

**Published:** 2022-05-24

**Authors:** Yunpeng Liu, Jing Dong, Ziqing Zhang, Yiqi Liu, Yang Wang

**Affiliations:** ^1^Department of Neurosurgery, Beijing Chao-Yang Hospital, Capital Medical University, Beijing, China; ^2^Department of Medical Engineering, Tsinghua University Yuquan Hospital, Beijing, China

**Keywords:** microbiota–gut–brain axis, ischemic stroke, risk factors, dysbiosis, probiotics

## Abstract

Ischemic stroke (IS) is among the top prevalent neurologic disorders globally today. Risk factors such as hypertension, diabetes, and aging, contribute to the development of IS, and patients with these risk factors face heavier therapeutic burden and worse prognosis. Microbiota–gut–brain axis describes the crosstalk between the gut flora, intestine, and center nervous system, which conduct homeostatic effects through the bacterial metabolites, the regulation of immune activity, also the contact with enteric nerve ends and vagus nerve. Nowadays, more studies have paid attention to the important roles that microbiota–gut–brain axis played in the risk factors of IS. In the current article, we will review the recent works focusing on the bi-directional impacts of gut dysbiosis and the pathogenic process of IS-related risk factors, for the purpose to summarize some novel findings in this area, and try to understand how probiotics could limit the development of IS *via* different strategies.

## Introduction

Ischemic stroke (IS) is one of the most common neurologic disorders in industrialized and developing countries (Favate and Younger, 2016; Pan et al., 2016; Takashima et al., 2017; Khan et al., 2019). According to the recent epidemiologic studies by the American Heart Association, the age adjusted death rate for stroke as an underlying cause of death was 37.1 per 100,000 in the USA, and a potential increase about 65% of stroke death is forecasted for the year 2030 (Virani et al., 2021). IS brings huge economic burden on the society and families, due to its severe symptoms at the onset, high expense during the treatment, and prolonged physical disability (Demaerschalk et al., 2010; Ma et al., 2021). During the past several decades, the recanalization therapy against AIS, i.e., thrombolysis and thrombectomy, were considered as efficient management to save the penumbral zone, thus to avoid the enlargement of the infarcted area (Jovin et al., 2015; Balodis et al., 2019). However, either the thrombolysis or the thrombectomy has limitations, which make it unsuitable for every patient. The time window of intervention is short, which must within the range of about 4.5–8 h directly after the onset of stroke. A significant proportion, 30–40% of patients, who underwent the therapy on-time, still suffered from so called “failed recanalization,” i.e., unsuccessful reconstruction of cerebral blood flow, and usually indicates a worse prognosis (Flottmann et al., 2018; Leischner et al., 2019). Thus, it is important to address the underlying mechanisms causing the persistent neuronal injury during the IS other than just removal of vessel occlusion.

The pathologic process of IS is complicated. The brain is not only suffered from the loss of oxygen and glucose, but also suffered from the secretion of damage associated molecular patterns (DAMPs) from de-oxygenized cells. DAMPs lead to direct death of neurons, injury of blood–brain barrier, alteration of endocrine system, and overreacted inflammation (Bustamante et al., 2016). Different types of cytokines and chemokines, released by injured tissue, could activate the resident microglia and recruit peripheral immune cells into the ischemic region, causing secondary damage on the penumbra (Gauberti et al., 2016). Adoptive immune components like T cells, migrated to the infarcted area in a later phase, and aid to change the local immune imbalance. Regulatory T cells (Tregs), to be specific, are the major immunosuppressive cell type that release anti-inflammatory cytokines like interleukin-10 (IL-10) and tumor growth factor β (TGF-β) (Xie et al., 2019; Santamaría-Cadavid et al., 2020). Overall, the neuroinflammation plays an important role either in the acute or chronic phase of IS, while clinically, there is still far from clear how the alteration of inflammatory statues would affect the general prognosis of IS patients. However, recent studies about microbiota–gut–brain axis and its correlation to chronic disorders do shed some lights on the future of IS management.

The microbiota–gut–brain axis describes the bi-directional communication between the brain and gastrointestinal tract. It is estimated that the human gut microbiota consists around 100 times of genes as that of human genome (Gill et al., 2006), and studies did find solid evidence that gut flora is connected with brain functions (Heijtz et al., 2011; Mohajeri et al., 2018; van Staaveren et al., 2021). The vagus nerve, for example, binds the brain directly with intestinal walls. The metabolites of gut bacteria, like short-chain fatty acid (SCFAs), could trigger the nerve plexuses in the intestinal wall, then sending signals through the ends of vagus nerve, to the solitary nucleus and finally reaches the diencephalon. On the other hand, the excitation of the vagus nerve could change the biological characters of gut flora in an efferent way, thus build a modulatory reflex (Forsythe et al., 2014). The peripheral immune system is also partly controlled by the microbiota–gut–brain axis, and various types of probiotics showed beneficial effects by restriction of overreacted immune responses (Fung et al., 2017). Furthermore, based on the existence of hierarchical structure of endocrine system and the feedback mechanism, various types of hormones, such as serotonin and corticosteroids, are demonstrated to be able to maintain the gut–brain homeostasis after neural injury (Stasi et al., 2019). Dysbiosis of gut flora has significant consequences for the host, especially affecting the nervous system during IS. Singh et al. (2016) identified reduced species diversity and bacterial overgrowth in middle cerebral artery occlusion (MCAO) model, and re-colonization by dysbiotic flora to germ-free mice led to increased ischemic volume after experimental stroke, suggesting an important role of a healthy bacterial composition in the normal progress of IS. Instead the direct linkages between IS and gut microbiota found in animal models of IS, it might be more interesting to explore how the microbiota could, in some cases, induce the onset of IS.

Various risk factors have been discovered of IS, including hypertension, diabetes/obesity, cardiac disorder and aging, etc. Many hints were unveiled either in clinic or laboratory about the relationships between these risk factors and the failed modulatory effects by microbiota-gut-brain axis, or, to be specific, the bi-directional effects of carrying the risk factors and gut dysbiosis. In this review, we will summarize the recent research articles related to the crosstalk between gut flora and brain infarction, with the focus mainly on the risk factors of IS, and possible pathogenic mechanisms that might lead to the onset of stroke. Moreover, we will also introduce some novel therapeutic opportunities toward the risk factors of IS based on the current understanding of microbiota–gut–brain axis.

## Hypertension

High blood pressure brings extra shear stress on the cerebral artery, and usually results in vessel injury and atherosclerosis, which are major pathologic process leading to IS. By comparison of microbiome in feces between people with normal blood pressure and hypertensive patients *via* 16S amplicon sequencing, Dan et al. (2019) found 54 differential genera between the hypertension group and control group. *Parabacteroides, Desulfovibrio*, and *Christensenella* showed higher abundance in either the hypertension group, or the patients with just systolic hypertension, probably due to the reduction of hydrogen sulfide (H_2_S), and following dampened inhibition of oxidation of butyrate and reduce energy supply of epithelium (Calderón-Pérez et al., 2021).

Direct linkage of metabolites from gut flora, to the hypertension and increased risk of stroke was demonstrated by Nie et al. Trimethylamine N-oxide (TMAO) is derived from the dietary choline and L-carnitine with assistance of gut microbiota. In this nested case–control study, hypertensive patients with higher level of TMAO had a 34% higher risk of stroke onset, compared to patients with lower level of TMAO (Nie et al., 2018). Moreover, spontaneous hypertensive stroke-prone (SHRSP) rats, which showed high incidence of stroke by 16 weeks of age, were found to have lower *Bacteroidetes*/*Firmicutes* ratio, if fed by high fat diet, indicating a tight correlation between diet, microbiota, and genetic background susceptible to high blood pressure and stroke (Singh et al., 2019).

An unhealthy lifestyle like the overconsumption of high-salt food is among the top several causes of hypertension. The 4-week continuous feeding of high-sodium diet in mice led to increase in the abundance of *Alistipes* and *Ruminococcaceae*, as well as reduction in the abundance of *Lactobacillus* (Zhang et al., 2019). The reduction of some bacteria species like *Bacteroides fragilis* by high-salt diet also led to higher secretion of intestinal-derived corticosterone, and the transplantation of feces from hypertensive mice fed by high-salt diet could lift up the receiver mice's blood pressure above normal, indicating the gut flora itself is able to modulate the blood pressure by modulation of hormone level (Yan et al., 2020). Furthermore, the changes of several types of SCFAs were found in the feces of rats fed by a high-salt diet; and the SCFAs also contributed to the development of hypertension (Bier et al., 2018; Chang et al., 2020). In a clinical study focusing on the stool metabolism changes in hypertensive patients, levels of acetate, butyrate propionate increased gradually from patients with normotension, borderline blood pressure, and hypertension, accompanied by taxa alterations of gut flora (Huart et al., 2019). By chronic oral treatment with butyrate-producing bacteria on spontaneously hypertensive rats, the increase in blood pressure and *Firmicutes*/*Bacteroidetes* ratio was prevented, and these effects might be mediated by the reduction of lipopolysaccharide (LPS)-Toll-like receptor 4 (TLR-4) pathway, and the infiltration of Tregs in the vasculature (Robles-Vera et al., 2020b). The same group also demonstrated that rats with deoxycorticosterone acetate (DOCA)-salt-induced hypertension had lower blood pressure after feeding with *Bifidobacterium breve*, and possible mechanisms beneath include improved colonic integrity, restored Th17 and regulatory T cells, and increased nitric oxide-dependent vasorelaxation (Robles-Vera et al., 2020a).

The involvement of neuroinflammation is discovered in patients with hypertension. Loss of normal neural microenvironment could facilitate the infarcted area to enlarge, and tight linkages were found between bowel inflammation and brain inflammation. Signals of gut dysbiosis are transmitted to the cardiovascular brain centers, i.e., paraventricular nucleus (PVN), rostral ventrolateral medulla (RVLM) and nucleus tractus solitaries (NTS), *via* the afferent fibers of the vagus nerve and circulating immune cells and cytokines (Wang et al., 2021). Stool samples collected from patients with inflammatory bowel disorders contained microbiota favoring gamma-aminobutyric acid (GABA) degradation. This microbiome change was consistent with a deficiency of GABAergic crosstalk between the mood center and PVN in the hypothalamus (Stevens et al., 2021). Kefir, a fermented milk drink, showed anti-hypertensive effects by changing the microbial composition in mouse by oral gavage (5% kefir grains in whole milk, 0.3 ml/100 g body weight), which was also correlated to reduced levels of IL-6 and tumor necrosis factor α (TNF-α), as well as attenuated microglial activation in the PVN and RVLM (de Almeida Silva et al., 2020). These evidences suggest that the gut microbiota could help to control the vessel constriction and inhibit the hypertension *via* its metabolites and the nervous system.

Another mechanism that gut microbiota helps to control hypertension is by changing the pharmacokinetics of antihypertensive drugs. Amlodipine, a member of calcium channel blocker, decreased and its metabolites increased when incubated with human and rat feces, and systemic exposure to amlodipine was elevated in rats treated with antibiotics, suggesting the reduced drug metabolic rate after the damage to normal flora (Yoo et al., 2016).

The intrauterine environment is demonstrated to affect the offspring for hypertension development in their adulthood (Edwards et al., 1993; Ojeda et al., 2008). In the rat model of systolic hypertension during pregnancy, Li et al. (2020) found maternal intake of captopril, a member of angiotensin-converting enzyme inhibitors (ACEIs), could modulate the constitution of the gut microbiome of both the dams and the pups by significant increases in *Corprococcus* and *Oscillospira*, and persistently lower systolic blood pressure. Interestingly, it was found that the maternal captopril feeding attenuated the microglia activation and neuroinflammation of the male offspring, suggesting the gender of the offspring might play a role in the sensitivity to the mother's intrauterine contact with ACEI (Gülke et al., 2018). On the other hand, the administration of the probiotic, *Lactobacillus casei*, in the pregnant dams was found successfully in attenuating the development of hypertension in offspring induced by high-fructose consumption (Hsu et al., 2018).

## Diabetes and Obesity

Diabetes and obesity are well-recognized risk factors for IS in both the elder (Meschia et al., 2014) and young populations (Mitchell et al., 2015). IS Patients with diabetes and/or obesity had higher odds for functional disability and overall worse prognosis (Park et al., 2019; Bailey et al., 2020). Similar finding was addressed by animal study, for MCAO rats with diet-induced obesity had larger infarcted area, smaller arterial lumen, and thicker vessel walls (Deutsch et al., 2009).

In diabetic mice with cerebral infarction/reperfusion injury, changing of microbiota by adding *Clostridium butyricum* showed positive effects, such as reduction of blood glucose level, amelioration of cognitive impairment, and attenuation of histopathologic changes in hippocampus (Sun et al., 2016). This study suggested that the specific strain of probiotics had benefits on neurologic behavior, as well as brain pathologic changes after stroke, with a possible target against diabetes, one of its major risk factors.

Due to the digestion and absorption mainly happen in the gastrointestinal tract, the gut microbiota is at the priority for sensing and responding to the nutrient components. Extra food intake would change the structure of gut flora, and the shifts of bacterial taxa might lead to obesity. Turnbaugh et al. demonstrated that mice with obesity had greater abundance of *Firmicutes* and increased ratio of it to *Bacteroidetes*, which were correlated with higher levels of acetate and butyrate, and lower calories contained in the caeca of obese mice. Interestingly, this kind of obesity could be adopted by fecal transplantation to WT mice (Turnbaugh et al., 2006). On the other hand, antibiotic treatment also affects the maintenance of energy balance. A 10% weight gain was found on patients with vancomycin treatment (Million et al., 2013). Chen et al., by adding engineered *E. coli* that highly express N-acylphosphatidylethanolamines (NAPEs) in the drinking water, successfully reduced the daily food intake of mice. They also found higher expression of genes encoding fatty acid oxidation in the gut microbiome, and reduced infiltration of monocytes/macrophages into liver (Chen et al., 2014). Another example is that the administration of *Lactobacillus plantarum* HACo1, isolated from fermented Korean kimchi, could reduce the mesenteric adipose depot and upregulate the lipid oxidative genes like leptin (Park et al., 2017). All the above studies suggest that a shifted microbiome, together with its metabolome, no matter triggered by diet or probiotics, could cause changes of the host's maintenance of energy balance.

The 3-hydroxy-3-methyl-glutaryl-coenzyme A reductase (HMG-CoA-reductase) inhibitors like statins are widely used for the treatment of hyperlipidemia. Statin treatment was found associated with lower prevalence of gut dysbiosis identified as lower Bact2 enterotype (Bacteroides2, an intestinal microbiota configuration with high *Bacteroides*/*Faecalibacterium* ratio and low microbial cell density), thus helps to maintain a normal gut–brain axis (Vieira-Silva et al., 2020). Statin sensitivity is also correlated with the gut flora biodiversity (Sun et al., 2018).

Similar findings were revealed in the research area of diabetes. Increased monocyte/macrophages, together with the expression of inflammatory cytokines were detected *via* the biopsy analysis of duodenal mucosa from patients with diabetes, and an increase of *Firmicutes*/*Bacteroidetes* ratio was found by 16S rRNA sequencing (Pellegrini et al., 2017). Treatment of prebiotics like non-digestible polysaccharide brought a faster 2-h glucose clearance in mice with high-fat diet, accompanied by a faster decline glucose level induced by exogenous insulin injection, and these evidences were correlated with a preserved bacterial diversity in the gut (Ahmadi et al., 2019). Another mechanism of increased glucose metabolism is through decreasing duodenal contraction. Fournel et al. (2017), by measuring the electrical duodenal activity *in vivo*, found that a bioactive peptide, apelin, could modify acetylcholine and nitric oxide release from neurons of enteric nervous system (ENS), led to raised insulin release, and upregulated the gene expression of type-4 glucose transporter. Interestingly, according to some studies, the microbiota composition of the offspring of patients with gestational diabetes was also changed, and early stage oligosaccharide consumption was positively correlated with the abundance of infant *Ruminococcus*, indicating the existence of a maternal microbial imprinting along glucose intake (Su et al., 2018; Wang et al., 2018; Ponzo et al., 2019).

Intestinal inflammation is known as a common complication of diabetes. Increase of pro-inflammatory cytokines and lesioned intestinal tissues are discovered in animals with diabetes. The ENS by this way could be affected and lost the reflex ability to modulate bowel constriction, thus building a malignant loop that facilitates the diabetes to develop. Feeding of probiotics could reverse this process by correction of the intestinal injury and maintenance of a normal ENS loop (Bessac et al., 2018). Besides of this, another beneficial neurological mechanism of probiotic on diabetic individuals is by the attenuation of synaptic injury. By feeding diabetic rats with a probiotic complex containing two types of *Lactobacillus* and one type of *Bifidobacterium*, Davari et al. (2013) found enhanced activation of superoxide dismutase and increased insulin in their circulation, and the electrophysiological results indicated the recovered basic synaptic transmission and restored hippocampal long-term potential (LTP).

## Aging

For elder populations, the incidence of IS could significantly rise up (Kapral et al., 2017; Khan et al., 2021). According to a recent epidemiologic study, stroke happened to 7.6% of adults aged 60 and above (Teh et al., 2018). Increased age is associated with a greater prevalence of vascular risk factors, as well as alterations of gut microbiota. The presences of *Bifidobacterium, Faecalibacterium*, and *Bacteroides* were found reduced in people above age 66, which were correlated with reduced fecal concentrations of SCFAs (Salazar et al., 2019). According to a study by Tan et al. (2021), the lack of SCFAs was linked to acute IS patients, and the level of SCFAs was negatively related to stroke severity and prognosis. This study might partly explain why elder patients with IS usually bear worse outcome. Also, the cognitive deficits in the elder IS patients were demonstrated to be a result of SCFAs' deficiency (Liu et al., 2020).

By using the experimental stroke model in aged C57BL/6 mice (18–20 months), Spychala et al. demonstrated increased *Firmicutes*/*Bacteroidetes* ratio in aged group. The fecal transplantation of young microbiota to elder mice leads to correction of gut microbiome, also positive changes in neurologic performances and survival rates, while the opposite transplantation, i.e., from aged mice to young mice, induced negative prognosis (Spychala et al., 2018). The SCFAs' effects on IS, which previously addressed by clinical studies, were also confirmed in animal works. Lee et al. (2020) demonstrated that not only the full young microbiome transplantation could attenuate symptoms in old MCAO mice, just a selected combination of SCFA producing bacteria strains, *Bifidobacterium longum, Clostridium symbiosum, Faecalibacterium prausnitzii*, and *Lactobacillus fermentum*, could alleviate poststroke neurological deficits. Possible explanation for SCFA's benefits on elder patients is the inhibition on the overproduction of reactive oxygen species (ROS) and nitric oxide (NO), and the prevention of the reduction of mitochondrial fusion gene expression (Hu et al., 2020).

Gut permeability was increased in the aged individuals, as what Qi et al. (2017) demonstrated that the serum concentration of zonulin, a marker of leaky gut, was 22% higher in the older adults as compared to young adults. Higher circulating zonulin was found to be a potential predictor of increased systolic blood pressure (Kim et al., 2017), as well as obesity and hyperlipidemia (Ohlsson et al., 2017). Probiotic administration showed protective effects on normal structures of intestinal epithelium. A human-origin probiotic cocktail containing 5 *Lactobacillus* and 5 *Enterococcus* strains was able to prevent leaky gut and inflammation in older mice (Ahmadi et al., 2020). Some probiotics like *Lactobacillus* could release polyphenol (Subrota et al., 2013), and polyphenol-rich diet on older people with leaky gut showed pronounced increase of the serum zonublin, together with reduction in blood pressure (Guglielmetti et al., 2020).

## Cardiac Disorders

Thrombus formation in the atrium brings a major source of embolus for cerebral artery and leads to IS. Patients with atrial fibrillation (AF) had global alterations of gut microbiome, including the overgrowth of *Ruminococcus* and *Streptococcus*, and the reduction of *Faecalibacterium* and *Alistipes*, etc., which were correlated with changed metabolic patterns in fecal and serum samples (Zuo et al., 2019). TMAO was also demonstrated to be able to facilitate the shortening effects on the effective refractory period of myocardium, and widening its window of vulnerability on mice with AF, possibly due to its function on cardiac autonomic nervous system (Yu et al., 2018).

In a hospital-based case/control study, patients with cardioembolic (CE) stroke had higher level of TMAO than patients with large artery atherosclerotic (LAA) stroke and healthy controls, suggesting a different metabolism pattern for stroke patients with cardiac origin (Xu et al., 2021). Moreover, distinct microbial composition was found between patients with CE and LAA stroke, for higher abundance of *Streptococcus* was demonstrated in the CE group, while the similar change was not seen in LAA group, compared to normal control. The functional and metabolic changes in patients with CE stroke were studied, for the microbial genes related to membrane transport and glycolipid metabolism showed increase, while the genes related to vitamin and glycan synthesis, as well as amino sugar and nucleotide sugar metabolism (Xu et al., 2020). All the evidence implied CE stroke did not share the same microbial characteristics with LAA stroke, and some bacteria like *Streptococcus* might conduct the pathogenic effects in CE stroke.

Cardiomyopathy predisposes to thrombus formation, and patients with cardiomyopathy had much higher incidences for IS occurrence (Crawford et al., 2004). However, few studies were found to focus on the direct crosstalk of cardiomyopathy and gut flora. The pathologic changes in myocardium might either be a direct or a subsequent change following the gut dysbiosis. Moreover, since viral infection to the myocardium could induce cardiomyopathy; works focusing on gut flora changes in patients with viral cardiomyopathy at early phases might be able to answer whether the gut flora management could slow down the anatomical change of the cardiac structure, thus reducing the risk of IS in the long-range.

Gut microbiota also contributes to the metabolic process of some cardiac drugs, thus indirectly affecting the process of cardiac disorders and the formation of thrombosis. Erythromycin and tetracycline treatment before and concurrent with digoxin could lead to reduced excretion of digoxin reduction products in urine and stool, followed by a two-fold increase of serum digoxin, which might induce cardiac arrhythmia (Lindenbaum et al., 1981).

## Smoking

Tobacco use contributes to the negative change of the immune system through damage of gut microbiota. Shanahan et al. (2018) discovered that either the previous or the current smokers had significantly reduced the bacterial diversity in the upper small intestinal mucosa, which was accompanied by more *Firmicutes, Rothia* and less *Prevotella*, and *Neisseria*. Maternal nicotine exposure *via* lactating showed decreased *Bacteroidetes*, increased *Firmicultes* and *Actinobacteria* in the feces of the offspring, indicating long-lasting effects of nicotine to change offspring's microbiota and eating behavior, which might predict extra calories intake and diabetes/obesity development (Rodrigues et al., 2021). Moreover, a recent study by Hosseini et al. (2021) demonstrated that the smoking-associated changes in microbiome were absent if the smoker quit smoke for more than 10 years, suggesting the necessity for smoking cessation for the purpose of maintaining healthy gut flora.

Nicotine not only causes tissue damage of lung itself, but also further breaks the integrity of intestine. Mis-homing of dendritic cells and T cells from injured lung to the intestinal epithelium, together with extra circulating IL-6, IL-13, and TNF-α, drives cross-organ inflammation (Gui et al., 2021). It is worth to mention that the nicotine's impacts on gut microbiota also follow a sex-specific manner. The oxidative stress and DNA repair genes were only enriched in nicotine-treated male mice. Multiple types of neurotransmitters, such as serine, aspartic acid, and glycine, were also only increased in the feces of male mice (Chi et al., 2017). However, not many articles nowadays focused on the direct connection between the nicotine exposure and the gut flora-induced neuroinflammatory alterations in IS patients, partly due to that smoking also behaves as a risk factor for cardiac disorders and hypertension, which might make the ‘smoking-gut dysbiosis-stroke' a rather difficult topic to study.

## Summary

**Figure 1:**
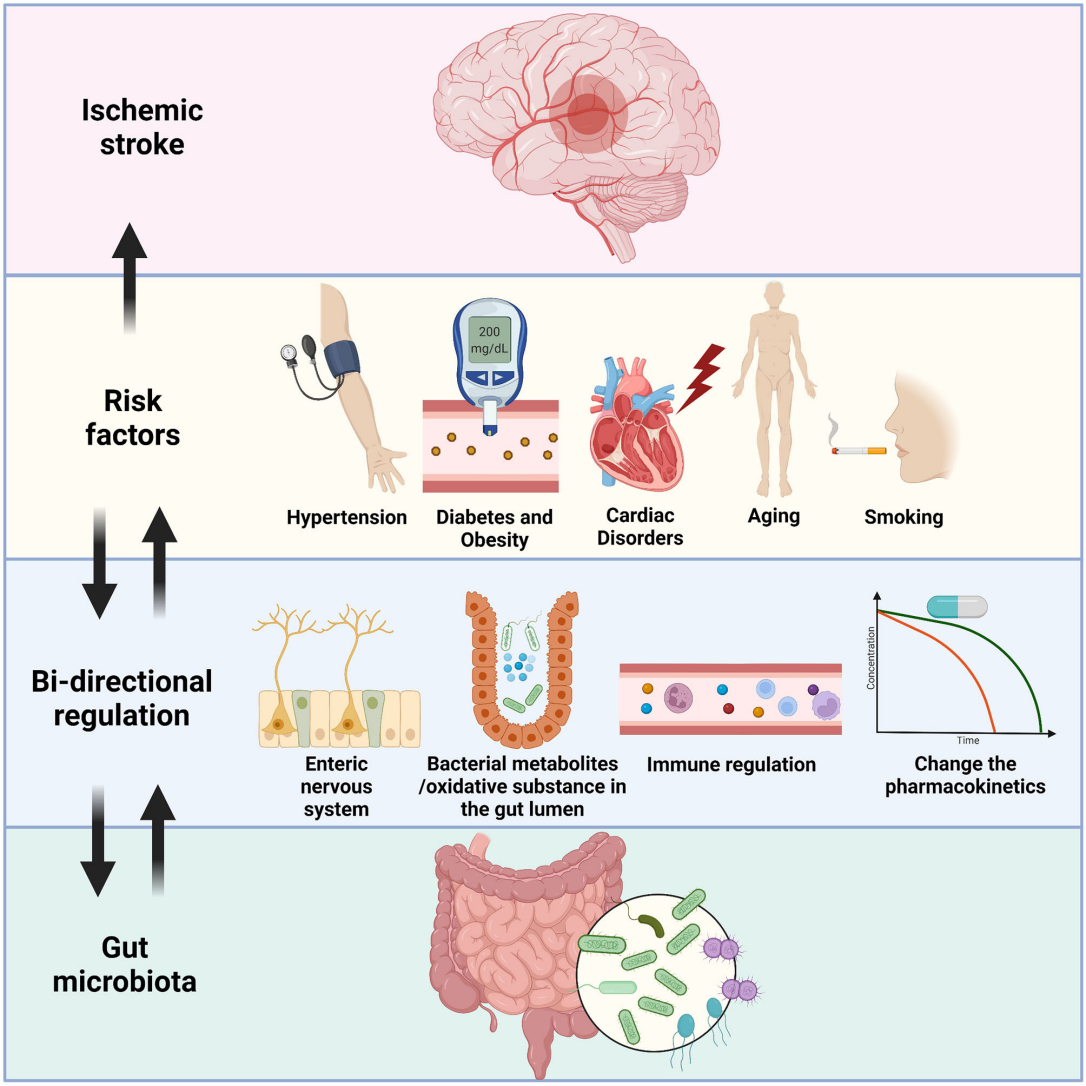
An illustration of the bi-directional relationship between gut microbiota and the risk factors of IS. Hypertension, diabetes/obesity, cardiac disorders, aging, and smoking are well-known risk factors of IS, and IS patients with these factors usually have unsatisfied prognosis. Gut flora could send signals through the enteric nervous system and reach specific brain areas like hypothalamus to control blood pressure and eating habit of the host. Some probiotics showed ability to lowering down the severity of these pre-stroke disorders by reduction of oxidative substances or secretion of short-chain fatty acids. Overreacted immune cells, including T cells, monocytes, and microglia were restricted by the administration of probiotics. Furthermore, the bacteria could also affect the pharmacokinetics of drugs, thus indirectly inhibit the development of diseases like hypertension. On the other hand, the gut dysbiosis is detected in the people who had these risk factors, which could fasten the general pathologic processes, and facilitate the onset of IS by similar mechanisms mentioned above.

In the current review, we summarized some recent findings relating to the crosstalk between the gut flora, the brain, and the risk factors of IS. Microbiota–gut–brain axis conducts multisystemic modulatory functions to attenuate the negative impacts of these risk factors had on the body. Through the degradation of extra oxidative substances, protection of intestinal epithelium, releasing SCFAs, metabolism of TMAO, inhibition of overwhelming immune injury, and transmission of signals *via* ENS and the vagus nerve, a healthy gut flora plays essential roles in the homeostatic maintenance in various risk factors of IS. Chronic metabolic diseases like hypertension and diabetes are tightly linked to gut dysbiosis by the reasons of nutrition and lifestyle. Probiotic treatment not only just change the microenvironment that limit the pathologic progress of these risk factors, but also conduct supplementary effect by facilitating the pharmaceutical treatment like calcium channel blocker and statin (all above mechanisms are briefly illustrated in Figure 1). The considerations imply further exploration on the gut microbiota management before and after the onset of IS, with possible routine monitoring of gut flora and lifestyle alterations, and specified clinical cares for IS patients with these gut dysbiosis related risk factors.

## Author Contributions

YuL, JD, and YW participated in idea conceptualization. YuL, ZZ, and YiL participated in the preparation of the manuscript. YuL participated in the editing of the manuscripts. All authors contributed to the article and approved the submitted version.

## Conflict of Interest

The authors declare that the research was conducted in the absence of any commercial or financial relationships that could be construed as a potential conflict of interest.

## Publisher's Note

All claims expressed in this article are solely those of the authors and do not necessarily represent those of their affiliated organizations, or those of the publisher, the editors and the reviewers. Any product that may be evaluated in this article, or claim that may be made by its manufacturer, is not guaranteed or endorsed by the publisher.
